# Motorcycle door crashes: An evaluation of crash characteristics in Taipei City, Taiwan

**DOI:** 10.1371/journal.pone.0208016

**Published:** 2018-12-27

**Authors:** Ping-Ling Chen, Ming-Heng Wang, Václav Linkov, Chih-Wei Pai

**Affiliations:** 1 Graduate Institute of Injury Prevention and Control, College of Public Health, Taipei Medical University, Taipei City, Taiwan; 2 Department of Traffic Management, Taiwan Police College, Taipei City, Taiwan; 3 Department of Traffic Psychology, CDV—Transport Research Centre, Brno, Czech Republic; Virginia Tech, UNITED STATES

## Abstract

**Objectives:**

Crashes due to motorcyclists colliding with an open car door can cause devastating injuries. In Taiwan, such crashes typically occur when the motorcyclist is travelling alongside a row of parallel-parked cars, and a driver suddenly opens the door in front of or next to the motorcycle without determining whether it is safe to do so. Injuries resulting from motorcycle door crashes tend to be severe. This study examined the factors that contribute to motorcycle door crashes.

**Methods:**

By using linked data from the National Traffic Crash Dataset and Emergency Medical Service for Taipei City for the years 2010–2015, this study estimated a mixed multinomial logit model to predict the likelihood of three types of door crashes: driver-door crashes, left passenger-door crashes, and right passenger-door crashes.

**Results:**

Data on 8237 motorcycle door crashes were extracted from the two datasets and matched. The results from the mixed multinomial logit model revealed that illegal parking, older car occupants, teenage car occupants, intoxicated car occupants and motorcyclists, and motorcycle speeding contribute to driver-door crashes; and female passengers and taxis as the type of vehicle involved in crash are associated with left passenger-door crashes.

**Conclusions:**

Our study suggested that controlling motorcycle speed, and prosecuting illegal parking and drink driving/riding may constitute effective countermeasures. The “Dutch Reach” intervention measure, which is commonly adopted in Europe for bicycle door crashes, should be applied in Taiwan to curb motorcycle door crashes, especially for elderly car occupants.

## Introduction

The motorcycle door crash, a unique crash type, involves a motorcycle colliding with the opening door of a car. Pai identified the motorcycle door crash as a car-motorcycle right-of-way (ROW) crash in which an inattentive car driver infringes upon the ROW of an approaching motorcyclist by opening the car door [1]. Door crashes are often caused by negligence–a driver’s or passenger’s failure to notice a motorcyclist travelling behind or on the adjacent lane. Crash impacts resulting from first contact with the door and/or second contact with the ground can be devastating. An opening door may cause motorcyclists to be thrown off the motorcycles, and the riders are likely to be run over by other vehicles, resulting in life-threatening injuries.

In Taiwan, traffic travels to the right side. The Liberty Times reported that between years 2010 and 2012, an average of five motorcyclists died and 1260 motorcyclists were injured due to door crashes annually in Taipei City [2]. By using data from Taiwan civil judgments in six cities on a total of 274 casualties resulting from door crashes, Huang reported that almost half of the motorcyclists involved (46%) were severely or fatally injured [3].

Studies on door crashes have primarily focused on bicycles and cars [4–7]. Findings from these studies emphasised the substantial injuries cyclists sustained as a result of a collision with an opened vehicle door [7, 8]. In a study by Pai, door crash was positively correlated with a one-way street, female cyclists, elderly cyclists, taxis, and dark hours [8]. Pai further highlighted the frequency of cyclist-car door crashes in built-up settings, attributing it to the likelihood of cars being more likely to be parked on the cycle lane. Johnson et al. reported that a majority of cyclists in such collisions were male, and the crashes commonly occurred on roadways with speed limits of up to 60km/h. Hunter and Stewart discussed the risk of door collision caused by improper bike lane placement next to on-street parking [9]. They pointed out that typical bicycle lanes are commonly located within the so-called “door zone” of parked automobiles, because a typical door extends to 3 to 3.5 feet, and bicycle lanes are often just slightly wider than that. Even when a bicycle travels at the centre of the cycle lane, cyclists are still susceptible to the risk of a door crash. Hunter and Stewart further advised that regardless of the presence of a cycle lane, cyclists should ride at least a door’s width from parked automobiles, which can improve sight triangles and increase bicyclists’ conspicuity from the perspective of a disembarking driver or passenger [9].

Most research focusing on car door crashes has been directed towards cyclists [7, 8]. Crashes in which motorcyclists collide with opening doors of cars have been rarely investigated. One possible reason for such a research gap is that although motorcycle door crashes are frequently accounted in traffic crash records, they may be classified more broadly (or misclassified) as a sideswipe or rear-end crash, leading to an underestimation of their prevalence. By using linked data from the Taiwan Traffic Crash Dataset and Emergency Medical Service (EMS) for Taipei City, this study estimated a mixed multinomial model to predict the likelihood of three door crash types (driver-door crash, left passenger-door crash, and right passenger-door crash).

The remainder of the paper is organised as follows. The next section describes the datasets analysed as well as the method used in the current research to evaluate characteristics of door crashes. Modelling estimation results, discussion, and implications of the findings are then provided in the subsequent sections.

## Method

### Data sources

The data sources in the present study were the National Traffic Crash Dataset and the EMS dataset. The National Traffic Crash Dataset is recorded by the National Police Agency, Taiwan. After every road traffic crash reported to police, qualified police crash investigators complete the report forms. Readers are referred to Chen et al. for a detailed description of the dataset [10]. Patients’ identification information that is used for linking the two datasets is encrypted by EMS Taipei. No individual patient or casualty can be identified and therefore, our study was exempted from review by an institutional review board (IRB#:n201510012).

Detailed information on which door was opened in motorcycle door crashes is not available in the National Traffic Crash Dataset. The EMS dataset (Taipei City) is a trauma-based dataset that is updated by emergency technicians and maintained by the Taipei City Fire Department. The dataset contains information such as vehicle attributes, crash location, exact time of the incident, and exact time of the arrival of an emergency technician at the scene. The EMS is free of charge to the public, and the dataset provides information on which door was opened (left front driver door, left back passenger door, right front passenger door, and right back passenger door). Personal IDs were used as an identifier for matching the two datasets on motorcycle door crashes.

**Fig 1:**
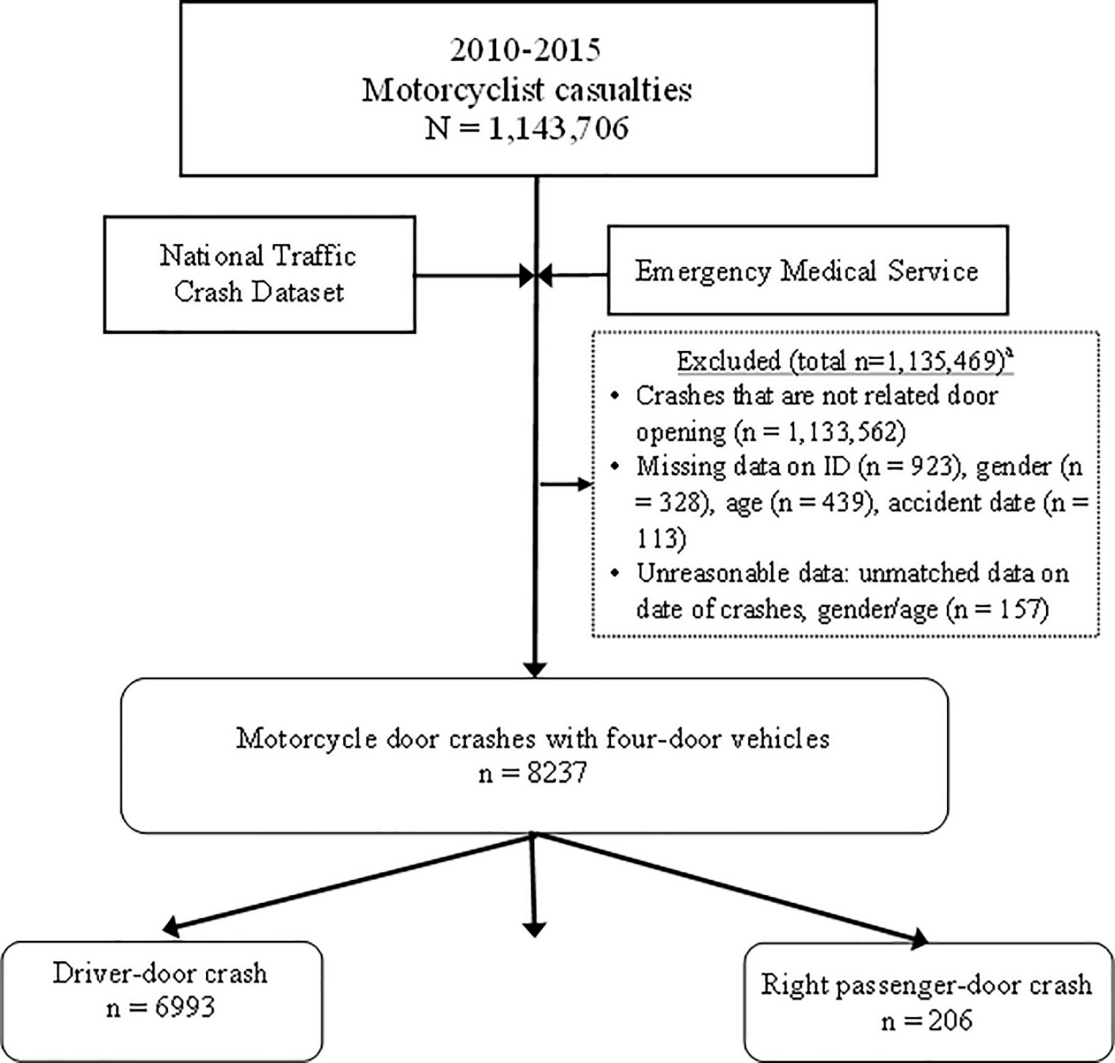
Study flow diagram.

The sample selection process from the two datasets is reported in Fig 1. The current research covered the data between 2010 and 2015. The study period was selected primarily because the EMS dataset for Taipei City becomes available from the year 2010, and the data for the year 2016 onwards are still not available yet.

As reported in Fig 1, data on 1,143,706 motorcyclist casualties caused by motorcycle crashes were extracted from the Taiwan Traffic Crash Dataset for the years 2010–2015. IDs were used as a key identifier for matching the police-reported data with the EMS dataset. Data with a missing ID, gender, age, and crash date were excluded. Unreasonable data (i.e., unmatched crash dates, genders or age between the two datasets) were also excluded. Data on a total of 8237 motorcyclist casualties were extracted from the records on motorcycle door crashes with four-door vehicles in Taipei City, of which 6993 cases (84.9%) involved the driver door, 1038 cases (12.6%) involved the left back passenger door, and 206 cases (2.5%) involved the right passenger door.

### Definition of variables

In this section, variables (e.g., demographic data, roadway characteristics, and vehicle and crash variables) examined in the analysis are defined in detail. Demographic data for the casualties included gender (male and female), car occupant and motorcyclist age (four groups: <18, 18–40, 41–64, and ≥65 years), alcohol use (yes: >0.03%; no: ≤ 0.03%), helmet use (yes or no), illegal parking (yes or no), type of vehicle involved in crash (car, taxi, or sports utility vehicle [SUV]), motorcycle speeding (yes or no), motorcycle engine size (moped: up to 50 cc, motorcycle: 51–250 cc, and heavy motorcycle: 251 cc or above), and location of the opened door (three levels: driver door, left passenger door, right passenger door). The variable “speed limit” was used to classify the roadway as urban (speed limit ≤ 50 km) or rural (speed limit ≥ 60 km). The urban/rural classification has been widely adopted in Taiwan (see the study of Liu et al. [11]): rural roadway is for 60km/h or above, and urban roadway is for 50km/h. The variable “street light condition” had three categories: daylight, street light lit in darkness, and unlit streets in darkness.

Alcohol use was evaluated on the basis of the blood alcohol consumption (BAC) level. According to Taiwanese law, drivers and riders with BAC > 0.03% are considered to be drink-driving, and a fine ticket is therefore issued to them. Drivers and riders with BAC > 0.03% who become involved in crashes are charged with an offense against public safety [12]. Those aged less than 18 years are generally identified as teenagers that cannot ride a motorcycle or drive a car legally. Those aged 65 years or above are generally identified as elderly people. A motorcycle was considered to be speeding when a police report indicated that the motorcycle exceeded the posted speed limit. Data on the right-front passenger door and right back door were combined due to a scarcity of data.

### Statistical analysis

The selected variables are classified by the three locations of the open doors in Table 1. This study investigated the contributory factors to the three door crash types (driver-door crash, left passenger-door crash, and right passenger-door crash). Because the dependent variable was multinomial (three door crash types), random parameters (mixed) multinomial regression models were used to examine the determinants of door crash types. Mixed multinomial regression models were estimated to account for unobserved heterogeneity that may arise from unmeasured variables such as risk perception, behavioural factors, and other socioeconomic factors that are not available in the datasets. An example of a behavioural factor is distraction by phone use. Car occupants using phones may be inattentive to approaching motorcyclists behind, which may lead to a crash. Ignoring the effect of such unobserved variables may lead to inconsistent estimates in non-linear models [13]. Therefore, random parameters models that allow for the possibility that the actors influencing motorcycle door crashes may vary across the observed crashes is essential. Chi-square independent test and Cramer’s V were estimated to assess multicollinearity among the variables. None of the variables were found to be correlated with each other.

**Table 1:** Descriptive statistics of selected variables (2010–2015).

Variable	Percentage		
	Driver-door crash	Left passenger-door crash	Right passenger-door crash
Fatality	3.4	1.2	0.2
Car occupant age (years) (<18/18-40/41-64/65 or above)	17.7/31.5/31.1/19.7	11.6/32.9/42.5/13.0	10.4/30.5/45.4/13.7
Motorcyclist age (years) (<18/18-40/41-64/65 or above)	36.3/22.7/19.6/21.4	24.7/31.2/22.3/21.8	25.0/29.8/30.2/15.0
Female car occupant	63.2	52.1	38.1
Female motorcyclist	38.2	45.9	56.7
Alcohol use (car occupant/motorcyclist)	13.2/11.0	5.6/7.4	2.3/3.5
Illegal parking	33.5	19.4	6.7
Type of vehicle involved in crash (car/taxi/SUV)	79.5/10.3/10.2	53.7/31.7/14.6	70.1/24.3/5.6
Motorcycle speeding	40.7	31.5	3.7
Motorcycle engine size (moped/motorcycle/heavy motorcycle)	24.7/63.5/11.8	36.1/46.6/17.3	52.8/34.6/12.6
Unhelmeted motorcyclists	2.7	1.4	1.2
Street light condition (daytime/lit streets in darkness/unlit streets in darkness)	56.7/27.6/15.7	60.9/28.9/10.2	62.3/29.8/7.9
Urban/rural roadways	89.6/10.4	94.7/5.3	99.6/0.4
**Total number of crashes**	6993	1038	206

In the present research, the utility of the door opened *i* for crash *n* is defined as follows:

$$U_{in}=\beta_{n}^{'}X_{in}+\varepsilon_{in}$$ Eq (1)

Where *X*_*in*_ is a vector of observed variables such as car occupant and motorcyclist attributes, vehicle characteristics, and environmental or temporal variables; β_*n*_ is a vector of parameters associated with *X*_*in*_; and ε_*in*_ is the error term. Assuming that β_*n*_ does not vary across the observed crashes in the standard multinomial logit formulation, the mixed logit model has β_*n*_ as a vector of estimable parameters for discrete outcome *n*, which varies across the observed crashes. The variation is observed with density *f(*β/θ*)*, where θ is a vector of parameters of the density distribution. In most applications, mixed models specify the density *f* to have a continuous distribution, such as a normal, lognormal (which restricts the effect of the estimated parameter to be positive or negative), triangular, or uniform distribution.

Given error terms that are independent and identically Gumbel distributed [14], the unconditional probability of one alternative *i* (from the set of all three door categories *I*) is the integral of the conditional probability with a multinomial logit form over parameter β of density *f*:

$$P_{in}=\int \left(\frac{\exp\left(\beta^{'}X_{in}\right)}{\sum_{I}\exp\left(\beta^{'}X_{in}\right)}\right)f\left(\beta |\theta \right)d\beta$$ Eq (2)

We first assume that all parameters are random and then evaluate their estimated standard deviations by using a zero-based (asymptotic) *t*-test for each parameter. A simulation-based maximum likelihood with 200 Halton draws was employed. Bhat suggested that 200 Halton draws provide accurate and precise maximum likelihood estimation [13].

Compared with the direct observation of the estimated parameters, reporting the marginal effect of each variable on the probabilities of the three door crash types is more informative. To identify the marginal effect of the *k*th indicator variable, we examined the change in estimated probability of the three crash types with a change in a variable from zero to one:

$$E_{X_{ik}}^{P_{in}}=\frac{P_{in}\left[\mathrm{given}\;X_{ik}=1\right]-P_{in}\left[\mathrm{given}\;X_{ik}=0\right]}{P_{in}\left[\mathrm{given}\;X_{ik}=0\right]}$$ Eq (3)

This is called the direct pseudo elasticity of the probability with respect to the explanatory variable. The model estimation results are reported in Table 2. The parameters in Table 2 are used in Eq (3) to calculate the average direct pseudo elasticity of the probability for each variable. The direct pseudo elasticities of the probabilities that aid the interpretation of the model results are displayed in Table 3.

**Table 2:** Mixed multinomial logit estimation results for three doors in motorcycle door crashes**.

Variable	Parameter	Standard error	*t*-value
Driver door			
Fixed parameter			
Constant	0.486	0.165	2.95
Teenage car occupant	0.160	0.071	2.25
Teenage motorcyclist	0.246	0.102	2.41
Female car occupant	0.433	0.103	4.20
Alcohol use by car occupant	0.753	0.137	5.50
Alcohol use by motorcyclist	0.301	0.134	2.25
Illegal parking	0.511	0.081	6.31
Motorcycle speeding	0.472	0.113	4.18
Unhelmeted motorcyclist	0.195	0.072	2.60
Unhelmeted and speeding	0.296	0.113	2.62
Unlit street condition in darkness	0.540	0.106	5.09
Car	0.257	0.080	3.21
Engine size (51–250 cc)	0.478	0.136	3.51
Random parameter			
Elderly car occupant (standard error)	0.827 (0.561)	0.163 (0.178)	5.07 (3.15)
Engine size (≥251cc) (standard error)	0.194 (0.131)	0.086 (0.053)	2.26 (2.47)
Left passenger door			
Fixed parameter			
Constant	0.305	0.117	2.61
Illegal parking	0.296	0.085	3.48
Unlit street condition in darkness	0.193	0.081	2.38
Motorcycle speeding	0.276	0.137	2.01
Female car occupant	0.133	0.063	2.11
Random parameter			
Taxi (standard error)	0.656 (0.413)	0.138 (0.117)	4.75 (3.53)
Restricted log-likelihood (constant only): -15,806 Log-likelihood at convergence: -10,973 ρ^2^ = 0.306			

** The right passenger-door crash is the baseline case with its parameters set at zero.

**Table 3:** Average direct pseudo-elasticities of the variables.

	Left front (driver door)	Left back	Right front/back
Teenage car occupant	34%	-6%	-6%
Teenage motorcyclist	57%	-5%	-5%
Alcohol use by car occupant	189%	3%	3%
Alcohol use by motorcyclist	102%	5%	5%
Illegal parking	196%	88%	-21%
Motorcycle speeding	128%	84%	7%
Unhelmeted motorcyclist	39%	-12%	-12%
Unhelmeted and speeding	88%	2%	2%
Unlit street condition in darkness	118%	53%	-6%
Elderly car occupant	227%	5%	5%
Engine size (51–250 cc)	125%	-18%	-18%
Engine size (≥251cc)	63%	-16%	-16%
Car	103%	-21%	-21%
Female car occupant	132%	96%	-7%
Taxi	-16%	139%	-16%
Female motorcyclist	-19%	-19%	77%
Engine size up to 50cc	-13%	-13%	96%

## Results

Table 2 presents the model estimation results for the mixed multinomial logit model of the types of door crashes. The right passenger-door crash is the baseline case with its parameters set at zero. The statistics in Table 2 reveal that the parameters have plausible sign and that the overall goodness of fit is fair (ρ^2^ = 0.306). The present study also estimated a traditional multinomial logit model; however, it is omitted here due to space limitations. A likelihood ratio test was performed to verify the applicability of the mixed multinomial logit model and to compare the results with the same specification estimated using the multinomial logit model. The test statistic is χ^2^=−2[LL*(*β_*R*_*)* −*LL(*β_*U*_*)*], where *LL(*β_*R*_*)* is the log likelihood at convergence of the restricted (traditional multinomial logit) model, and *LL(*β_*U*_*)* is the log likelihood at convergence of the ‘unrestricted’ (mixed logit) model. This statistic is χ^2^ distributed, with degrees of freedom equal to the difference in the number of parameters between the restricted and unrestricted models. This results in an χ^2^ statistic of 312.6, and with 5 degrees of freedom (the multinomial logit model yields a log-likelihood at convergence of 11327.2 with 20 parameters). This gives a confidence level of 99.99% indicating that we are more than 99.99% confident that the mixed logit model is statistically superior.

For specifying the type of the distribution, various distributions were assumed to determine proper densities for random parameters. Parameters were assumed to have linear effects across the observations if the estimated standard errors were not statistically different from 0. Random parameters were those that produced statistically significant standard errors for their assumed distributions. The uniform distribution appears to provide the best statistical fit.

Looking at the specific results in Tables 2 and 3, the parameters for teenage car occupants and motorcyclists (<18) are fixed and positive, implying that teenage car occupants and motorcyclists are associated with an increased probability of driver-door crashes (34% and 57%, respectively; Table 3). In Taiwan, the legal age for driving a car and riding a motorcycle is 18 years, and the present study finding is in agreement with studies that have reported that travelling unlicensed is associated with an increased crash risk [15, 16]. The current research adds to the existing motorcycle safety literature by demonstrating that teenage car occupants without a lawful driving license may fail to notice an approaching motorcyclist and consequently strike the motorcyclist by opening the door.

Alcohol use is a crucial variable influencing driver-door crashes. Alcohol use by either car occupants or motorcyclists was found to be associated with an increased probability of driver-door crashes (189% and 102% respectively). This result can be explained by the fact that alcohol use impairs the ability to rapidly focus vision and process information, and lowers alertness. Illegal parking appears to be a significant determinant of driver-door and left passenger-door crashes. The results reveal that illegal car parking increased the probability of driver-door and left passenger-door crashes (196% and 88%, respectively; Table 3).

The estimated parameter for motorcyclist speeding appears to be positive and fixed across the observations. Those speeding were more likely than those not speeding to be involved in driver-door and left passenger-door crashes (128% and 84%, respectively; Table 3). We attribute this finding to the possibility that car drivers are less able to notice a motorcyclist who is speeding from behind, and therefore open the door.

Motorcyclists riding unhelmeted appear to be a contributory factor to driver-door crashes, with an increased probability by 39% (Table 3). We hypothesised that those riding unhelmeted tend to engage in risk-taking behaviours. Here, one interaction term for the effects “unhelmeted riding” and “speeding” was added to the model specification. The results reveal that those travelling unhelmeted and speeding simultaneously, compared with those travelling helmeted and not speeding, were more likely to be involved in driver-door crashes (88%; Table 3).

An unlit street condition in darkness was found to result in an increased probability of driver-door and left passenger-door crashes (118% and 53%, respectively; Table 3), and the effect appears to be fixed across the observations. The explanation for this effect is intuitive: on unlit streets in darkness, car occupants may experience additional difficulty noticing oncoming motorcycles from behind.

Elderly car occupants were found to be more likely to be involved in driver-door crashes (227%; Table 3) than the other age groups. The parameter for this particular age group appears to be random, with a uniform distribution. The non-linear effect on driver-door crashes may be attributed to the possibility that although older drivers may exhibit decreased cognitive ability to detect an oncoming motorcycle, they can be experienced drivers and consequently open the door more cautiously.

Two groups of motorcyclists, namely engine size 51–250 cc and 251 cc or above, were more likely to be involved in driver-door crashes (125% and 63%, respectively; Table 3). The effect of an engine size of 51–250 cc appears to be linear, whereas that of a greater size (251 cc or above) appears to be heterogeneous. The non-uniform effect of a greater engine size can be attributed to two factors: larger motorcycles travel faster, giving car occupants less time to notice them; and larger motorcycles may be owned by more experienced riders who may be more capable of taking evasive actions in an event that a door is opened.

The estimated parameter for the indicator variable for cars was positive and fixed across the observations. Car occupants were more likely than occupants of other vehicle types to be involved in driver-door crashes (103%; Table 3). Taxis contributed to left passenger-door crashes (139%; Table 3). This result may be due to passengers often alighting from the left passenger door. Note that alighting from the left passenger door violates traffic rules. This finding underscores the importance of prosecuting taxi passengers alighting from the left passenger side.

Compared with male car occupants, female car occupants contributed more to driver-door and left passenger-door crashes (132% and 96%, respectively; Table 3). Notably, in door crashes involving female motorcycle riders, an increase in the probability of right passenger-door crashes (77%) was observed. Moped riders exhibited an increased probability of right passenger-door crashes (96%; Table 3). Such an effect is likely to attribute to travel pattern of mopeds: it is common in Taiwan that mopeds travel on roadway shoulder and therefore exhibits higher risks of right passenger-door crashes.

## Discussion and conclusions

The current research contributes to the growing literature related to motorcycle safety, and fills the major gap in extant literature by examining risk factors affecting motorcycle door crashes. By using linked data from the National Traffic Crash Dataset and the EMS dataset, our descriptive analyses firstly show that driver-door crashes account for 85% of the all motorcycle door crashes, with the highest fatality rate (3.4%, Table 1). Evidently, driver-door crashes pose a considerable threat to motorcyclist safety in terms of their frequencies and crash consequences. Intervention points targeting driver-door crashes should therefore be prioritised. Our estimation results of mixed multinomial logit models revealed several crucial determinants of driver-door crashes, including elderly car occupants, young car occupants, alcohol use by car occupants or motorcyclists, unlit street condition, motorcycle speeding, and illegal parking. These results are discussed below.

Studies have well documented that motorcycle ROW crashes are primarily due to car drivers not seeing the motorcycle (i.e., detection failure) or misjudging the speed or distance (i.e., decision error) of the motorcycle [1]. It is possible that motorcycle door crashes similarly involve a car driver failing to identify a motorcycle or accurately judge its speed or distance. Investigating whether detection or decision failure individually (or together) leads to motorcycle door crashes is outside the scope of this study. However, our study has uncovered several crucial determinants of driver-door crashes. First, elderly car occupants were associated with an increased probability of driver-door crashes. Studies [17] have consistently suggested that elderly drivers were over-involved in motorcycle ROW crashes at intersections, and the current research adds to motorcycle safety research by demonstrating that elderly car occupants play a role in driver-door crashes. Evidently, older motorists’ age-related impairment and cognitive decline contribute to not only motorcycle ROW crashes at intersections [1] but also motorcycle door crashes (driver-door crashes).

Another age-related effect is that of teenage car occupants–the likelihood of a driver-door crash tends to increase when the car occupant is a teenager. This finding can be explained by teenage car occupants being unlicensed drivers. Their navigating ability in traffic may not be as advanced as that of licensed drivers; thus, they may open the door recklessly. Coupled with the finding that elderly car occupants are associated with an increased likelihood of driver-door crashes, this research points to an urgent need to target these two age groups when considering intervention points such as education programmes and driving licensure tests for teenagers.

Teenage motorcyclists appear to contribute to driver-door crashes, which may, similar to the effect of teenage car occupants, be attributed to their unlawful and inexperienced riding. In addition, the interaction effect of teenage motorcyclists and speeding appears to be a crucial determinant of driver-door crashes. This interaction effect likely captures the effect of teenage riders’ risk-taking behaviours on driver-door crashes.

When either the car occupant or motorcyclist was identified to be intoxicated, the likelihood of a driver-door crash increased. Research [18] has suggested that driver’s ability to detect an oncoming motorcycle from behind can be impaired by alcohol use. Certainly, prosecuting drink and driving may constitute an effective countermeasure for reducing motorcycle crashes in general and motorcycle door crashes in particular.

An unlit street condition in darkness was identified to contribute to driver-door crashes. Studies have suggested that improving motorcycle’s conspicuity through, for instance, manipulating headlights, may reduce detection or decision failure by car drivers [19, 20]. The current study suggested that motorcyclists should increase their alertness level when travelling parallel to parked vehicles, especially in unlit street conditions in darkness.

Motorcycle speeding was found to be an important factor to driver-door crashes. Research [1, 18] has reported that motorcycle excessive speed would induce driver’s speed and/or judgement errors at intersections where a turning motorists was in a need to intersect with an approaching motorcycle. It is an important finding that such a motorcycle-speeding effect applies not only to motorcycle crashes at intersections but also to motorcycle door crashes. The current study suggested that while motorcycles increase their alertness level when travelling parallel to parked vehicles, their speed management may also play a crucial role in reducing door crashes.

Research [21] has pointed out that on-street parking was associated with elevated crash risks. Illegal parking in general results in restricted roadway space, thereby increasing door crash risks. Our study finds that illegal parking was associated with a higher risk of driver-door crashes. Prosecuting illegal parking is beneficial for all road users’ road safety in general, and for reducing motorcycle door crashes in particular.

Taxis were found to contribute to left passenger door crashes. The current traffic regulations in Taiwan require that all taxi passengers alight from the right passenger door (i.e., the kerbside). This finding underscores the importance of prosecuting taxi passengers alighting from the left side.

A widely adopted cyclist safety intervention measure in the Netherlands is the Dutch Reach [22], in which passengers and drivers are required to open the car door using the far hand, thereby turning their body and thus ensuring that they can view the traffic behind. Since year 2013, the Dutch Reach has been regulated in the licensure test in Taiwan. The latest test rule that has begun since year 2016 is that those not obeying the Dutch Reach fail their driving test. In addition to being evaluated in licensure test, the Dutch Reach should be promoted among all passengers and drivers in general and to certain groups such as elderly car occupants in particular.

The present study has a few intrinsic limitations. The most salient research limitation stems from the unavailability of variables in the two databases. Although the National Traffic Crash Dataset and the EMS dataset are a rich source of some variables, several other crucial factors are not readily available, including, motorcycle speed, geometric factors, and other rider and car occupant attributes (experience, education level, and fitness to ride), all of which have been reported to affect motorcycle ROW crashes [1]. Similarly, these factors may also affect motorcycle door crashes in which a motorcycle’s ROW is violated by car occupants opening the door. Detailed data (e.g., behavioural mechanism and intention data that can be collected only through an observational study and a questionnaire survey) would provide additional insights into motorcycle door crashes.
